# Severe Hyperkalemia Caused by Excessive Consumption of Dried Orange Juice in a Patient With Normal Renal Function

**DOI:** 10.7759/cureus.104381

**Published:** 2026-02-27

**Authors:** Omer Elshaikh, Osama Elkhider, Alisha Sharma

**Affiliations:** 1 Pathology and Laboratory Medicine, NEOM Hospital Operated by Fakeeh Care, Sharma, SAU; 2 Internal Medicine, Medical College of Wisconsin, Milwaukee, USA; 3 Internal Medicine, VCU Medical Center North Hospital, Richmond, USA

**Keywords:** dietary potassium intake, dried orange juice, electrocardiographic changes, hyperkalemia, normal renal function

## Abstract

Hyperkalemia is a potentially life-threatening electrolyte abnormality most commonly associated with impaired renal excretion, medication effects, or metabolic disturbances. Severe hyperkalemia resulting from dietary potassium intake alone is uncommon in individuals with preserved renal function due to normally effective renal excretion mechanisms. We report a case of a 40-year-old man with no known past medical history who presented with progressive neuromuscular symptoms, palpitations, and syncope and was found to have life-threatening hyperkalemia with serum potassium levels rising to 9.4 mmol/L and characteristic electrocardiographic changes, including peaked T waves and QRS widening. Renal function, acid-base status, and medication history were unremarkable, and pseudohyperkalemia was excluded. The patient required intensive care unit admission and was treated with intravenous calcium, insulin with dextrose, beta-agonist therapy, and potassium binders, resulting in prompt clinical and biochemical improvement without the need for renal replacement therapy. Further history revealed excessive daily consumption of large volumes of a dried orange juice powder reconstituted as a beverage over several weeks. Discontinuation of the potassium-rich beverage led to sustained normalization of serum potassium levels and complete resolution of symptoms. This case highlights that extreme and sustained intake of potassium-rich liquid dietary sources can overwhelm physiologic compensatory mechanisms and cause severe hyperkalemia even in patients with normal renal function. A thorough dietary history is essential when evaluating unexplained hyperkalemia to ensure timely diagnosis and prevent potentially fatal complications.

## Introduction

Hyperkalemia, defined as a serum potassium concentration above 5.0 mmol/L, can result in significant cardiac and neuromuscular complications, including fatal arrhythmias [1,2]. Potassium homeostasis is primarily regulated by renal excretion, with approximately 90-95% of daily potassium intake eliminated through distal nephron secretion under aldosterone regulation, allowing maintenance of serum potassium within a narrow physiologic range [1,2]. Disruption of this tightly regulated process may lead to hyperkalemia, which can manifest with neuromuscular symptoms such as weakness and paresthesia as well as characteristic electrocardiographic changes, including peaked T waves and QRS widening [1,2].

In most cases, hyperkalemia arises from decreased renal potassium excretion, use of potassium-retaining medications, metabolic acidosis, or conditions associated with increased cellular potassium release [1]. In contrast, isolated dietary potassium excess is an uncommon cause of severe hyperkalemia in individuals with normal renal function, as renal potassium excretion typically adapts efficiently to increased intake [2,3]. Nevertheless, extreme or sustained potassium consumption, particularly in liquid form, may overwhelm compensatory mechanisms. We present a case of severe hyperkalemia caused by excessive intake of dried orange juice in a patient without underlying renal disease.

## Case presentation

A 40-year-old man presented to the emergency department with a three-week history of progressive dyspnea, palpitations, generalized fatigue, paresthesia, and muscle weakness. He also reported multiple episodes of near-syncope. He denied chest pain, fever, gastrointestinal symptoms, or recent illness.

The patient had no known medical conditions and was not taking prescription medications, over-the-counter drugs, or herbal supplements. He denied alcohol or illicit drug use. Physical examination was unremarkable, with stable vital signs and no focal neurologic deficits.

Initial laboratory testing revealed severe hyperkalemia with a serum potassium concentration of 8.5 mmol/L, which increased to 9.4 mmol/L on repeat testing from separate blood samples. There was no evidence of hemolysis. Renal function was preserved, with a serum creatinine of 0.9 mg/dL and an estimated glomerular filtration rate of 88 mL/min/1.73 m². Acid-base status was normal, with a venous pH of 7.40 and a bicarbonate level of 25 mmol/L. Complete blood count and liver function tests were within normal limits. Selected laboratory values on presentation are summarized in Table 1.

**Table 1:** Laboratory Values on Presentation

Laboratory Test	Result	Reference Range
Potassium, initial (mmol/L)	8.5	3.5-5.1
Potassium, repeat (mmol/L)	9.4	3.5-5.1
Sodium (mmol/L)	138	135-145
Chloride (mmol/L)	100	98-108
Bicarbonate (mmol/L)	25	23-32
Blood urea nitrogen (mg/dL)	16	8-23
Creatinine (mg/dL)	0.9	0.60-1.10
eGFR (mL/min/1.73 m²)	88	>60
Blood glucose (mg/dL)	109	80-140
Venous pH	7.40	7.32-7.42
AST (U/L)	25	10-41
White blood cell count (×10⁹/L)	7.5	4.0-11.0

According to the medical record, electrocardiography demonstrated findings consistent with severe hyperkalemia, including peaked T waves, PR prolongation, absent P waves, and QRS widening. The original electrocardiographic tracing could not be retrieved from institutional records.

Given the degree of hyperkalemia (9.4 mmol/L) and associated electrocardiographic changes, the patient was admitted to the intensive care unit and treated with intravenous calcium gluconate (1 g), regular insulin (10 units intravenously) with 25 g of dextrose, nebulized albuterol, and potassium binders. Renal replacement therapy was not required.

During further history-taking, the patient reported consuming more than 6 L per day of a dried orange juice powder reconstituted as a beverage. According to the product’s nutritional label, each 12 g serving contained approximately 145 mg of potassium and was intended to be mixed with 4 oz (120 mL) of water, corresponding to approximately 1,200 mg of potassium per liter. Based on the reported intake, the patient’s estimated daily potassium consumption exceeded 7,000 mg. He stated that he had initiated this practice to reduce caffeine intake and had been consuming the beverage compulsively for several weeks. Following discontinuation of the potassium-rich beverage and continued medical management, serum potassium levels gradually declined and stabilized at 3.5 mmol/L. The patient’s symptoms resolved completely, and follow-up laboratory testing after discharge confirmed sustained normokalemia.

## Discussion

In individuals with intact renal function, dietary potassium intake alone is an uncommon cause of severe hyperkalemia because renal potassium excretion typically increases in response to elevated intake and maintains potassium homeostasis [1,2]. Approximately 90-95% of dietary potassium is eliminated by the kidneys through distal nephron secretion under aldosterone regulation, allowing serum potassium to remain within a narrow physiologic range [1,2]. However, exceptionally large and sustained potassium loads, particularly in liquid form, allowing rapid gastrointestinal absorption, may transiently exceed the kidney’s short-term excretory capacity and result in clinically significant hyperkalemia [2].

The recommended adequate intake of potassium for adults is approximately 2,600-3,400 mg per day, depending on age and sex [4]. Based on the product’s nutritional label, each 12 g serving of the dried orange juice powder contained approximately 145 mg of potassium and was intended to be mixed with 4 oz (120 mL) of water, corresponding to approximately 1,200 mg of potassium per liter. Given the reported intake of more than 6 L per day, the patient’s estimated daily potassium consumption exceeded 7,000 mg, more than double the recommended adequate intake. Although no specific upper limit has been established for potassium intake in healthy individuals, extreme and sustained consumption of potassium-rich foods or beverages may overwhelm physiologic compensatory mechanisms.

The estimated potassium intake was calculated based on the manufacturer’s recommended preparation instructions. However, the exact concentration of the beverage consumed by the patient could not be verified, and it is possible that additional powder was used per serving, potentially resulting in even greater potassium exposure.

Dried orange juice and orange juice concentrates are well-recognized dietary sources of potassium, and cases of hyperkalemia associated with excessive orange juice consumption have been reported, particularly in individuals with impaired potassium excretion, such as those with chronic kidney disease [3]. Previous case reports have also described hyperkalemia associated with excessive intake of potassium-rich foods or beverages, including dried fruits, bananas, and orange juice, often in the setting of behavioral or psychiatric conditions or compulsive ingestion behaviors [3,5-7]. Notably, Berk et al. reported severe hyperkalemia in a patient with schizophrenia and normal renal function following excessive orange juice consumption, highlighting the role of behavioral factors in precipitating electrolyte disturbances even in the absence of intrinsic kidney disease [8]. These findings support the possibility that extreme dietary intake alone can overwhelm renal potassium handling even in patients with preserved renal function.

Alternative causes of hyperkalemia were carefully considered in this patient. Pseudohyperkalemia was excluded through repeated measurements without hemolysis, normal AST levels, and stable blood counts [9]. Renal failure, metabolic acidosis, medication-induced hyperkalemia, adrenal insufficiency, and rhabdomyolysis were not supported by clinical or laboratory findings. Hyperkalemic periodic paralysis was also considered as a potential contributing factor; however, this diagnosis was unlikely given the absence of a prior history of episodic weakness, lack of family history, progressive rather than episodic symptoms, and sustained severe hyperkalemia that resolved following cessation of excessive dietary potassium intake.

The management of severe hyperkalemia focuses on stabilization of the cardiac membrane, intracellular potassium shifting, and enhancement of potassium elimination. Intravenous calcium is administered to stabilize myocardial excitability, while insulin with dextrose and beta-agonists such as albuterol promote intracellular potassium redistribution. Potassium elimination may be achieved through diuretics, potassium-binding agents, or renal replacement therapy in refractory cases. In this patient, prompt medical therapy resulted in rapid clinical and biochemical improvement without the need for dialysis.

This case adds to the existing literature by demonstrating that life-threatening hyperkalemia can occur in otherwise healthy individuals when potassium intake from concentrated dietary sources is extreme and sustained. It underscores the importance of obtaining a thorough dietary history when evaluating unexplained hyperkalemia, even in patients with preserved renal function.

A limitation of this case is that the original electrocardiographic tracing was not available for publication despite attempts to retrieve it from institutional records, and electrocardiographic findings were reported based on medical record documentation.

## Conclusions

Severe hyperkalemia can occur as a result of extreme consumption of potassium-rich beverages such as dried orange juice, even in patients with normal renal function. This case demonstrates that sustained intake of large quantities of liquid potassium sources can overwhelm physiologic renal compensatory mechanisms. Clinicians should maintain a high index of suspicion and obtain a thorough dietary history when evaluating unexplained hyperkalemia. Early recognition and prompt treatment are essential to prevent potentially fatal complications.
